# The added value of multi‐state modelling in a randomized controlled trial: The HOVON 102 study re‐analyzed

**DOI:** 10.1002/cam4.4392

**Published:** 2021-12-24

**Authors:** Katerina Bakunina, Hein Putter, Jurjen Versluis, Eva A. S. Koster, Bronno van der Holt, Markus G. Manz, Dimitri A. Breems, Bjorn T. Gjertsen, Jacqueline Cloos, Peter J. M. Valk, Jakob Passweg, Thomas Pabst, Gert J. Ossenkoppele, Bob Löwenberg, Jan J. Cornelissen, Liesbeth C. de Wreede

**Affiliations:** ^1^ Department of Hematology HOVON Data Center Erasmus MC Cancer Institute Rotterdam The Netherlands; ^2^ Department of Biomedical Data Sciences Leiden University Medical Center Leiden The Netherlands; ^3^ Department of Hematology Erasmus University Medical Center Cancer Institute Rotterdam The Netherlands; ^4^ Department of Hematology Leiden University Medical Center Leiden The Netherlands; ^5^ Department of Medical Oncology and Hematology University Hospital Zurich Zurich Switzerland; ^6^ Department of Hematology Hospital Network Antwerp Stuivenberg/Middelheim Antwerp Belgium; ^7^ Department of Internal Medicine Hematology section Haukeland University Hospital Bergen Norway; ^8^ Department of Clinical Science University of Bergen Bergen Norway; ^9^ Department of Hematology Amsterdam UMC VU University Medical Center Cancer Center Amsterdam Amsterdam The Netherlands; ^10^ Department of Hematology University Hospital Basel Basel Switzerland; ^11^ Department of Medical Oncology University Hospital/Inselspital Bern Switzerland

**Keywords:** AML, clofarabine, current leukemia‐free survival, HSCT, multi‐state model, RCT

## Abstract

Clofarabine is an active antileukemic drug for subgroups of patients with acute myeloid leukemia (AML). Multi‐state models can provide additional insights to supplement the original intention‐to‐treat analysis of randomized controlled trials (RCT). We re‐analyzed the HOVON102/SAKK30/09 phase III RCT for newly diagnosed AML patients, which randomized between standard induction chemotherapy with or without clofarabine. Using multi‐state models, we evaluated the effects of induction chemotherapy outcomes (complete remission [CR], measurable residual disease [MRD]), and post‐remission therapy with allogeneic stem cell transplantation [alloSCT] on relapse and death. Through the latter a consistent reduction in the hazard of relapse in the clofarabine arm compared to the standard arm was found, which occurred irrespective of MRD status or post‐remission treatment with alloSCT, demonstrating a strong and persistent antileukemic effect of clofarabine. During the time period between achieving CR and possible post‐remission treatment with alloSCT, non‐relapse mortality was higher in patients receiving clofarabine. An overall net benefit of treatment with clofarabine was identified using the composite endpoint current leukemia‐free survival (CLFS). In conclusion, these results enforce and extend the earlier reported beneficial effect of clofarabine in AML and show that multi‐state models further detail the effect of treatment on competing and series of events.

## Graphical abstract

Re‐analysis of an RCT with more advanced statistical method ‐ multi‐state models, gives additional insights to classical trial analysis methods.

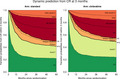

## 1 INTRODUCTION

Active antileukemic activity by clofarabine has been demonstrated in acute myeloid leukemia (AML) patients, but its impact on long‐term survival has been less clear.^1^, ^2^, ^3^, ^4^, ^5^, ^6^, ^7^, ^8^, ^9^, ^10^, ^11^, ^12^, ^13^ We recently reported the results of a prospective randomized phase III trial (HOVON102AML/SAKK30/09), showing that clofarabine reduces relapse rates and may improve event‐free survival (EFS), the latter being restricted to the subgroup of intermediate risk AML patients.^10^ Our study and other phase III studies in cancer often select long‐term survival endpoints such as EFS or overall survival (OS) for the primary efficacy analysis. However, long‐term clinical outcomes are determined by series of treatments rather than only the treatment given at onset. For example, the application of different post‐remission treatments in AML may hamper a straightforward evaluation of drugs used in induction treatment.^9^ Moreover, post‐remission treatment is considered based on the risk of relapse determined by the genetic profile of the AML and presence of measurable residual disease (MRD), and counterbalancing non‐relapse mortality (NRM) risk, which further complicates the analysis.^14^, ^15^, ^16^ Therefore, to disentangle the effects of various treatments, more advanced statistical methodology are needed.^1^, ^2^, ^3^

Intention‐to‐treat (ITT) analysis is the gold standard in randomized controlled clinical trials (RCT) since it provides a valid overall evaluation of the efficacy of a treatment regimen. However, ITT does not consider any intermediate events––either treatments or clinical events, such as achievement of MRD. Several standard survival analysis methods are available that attempt to take time‐dependent treatments into account: (a) censoring survival outcomes at the time of treatment initiation; (b) using treatment initiation as a time‐dependent covariate in a Cox regression model; (c) landmarking, where the groups (with or without treatment) are defined based on the treatment allocation before the landmark time; but they all have considerable limitations. Multi‐state models have been introduced several decades ago but have gained increased interest recently. These models put long‐term survival outcomes and intermediate outcomes and treatments into one framework, which allows to evaluate the effect of a sequence of events on the long‐term outcomes. Therefore, we set out to re‐analyze relapse and death in a recent HOVON102/SAKK30/09 study evaluating clofarabine as induction treatment in AML, by taking the outcomes of remission‐induction therapy and post‐remission treatment with allogeneic stem cell transplantation (alloSCT) into account.

The re‐analysis of a recently published RCT using the multi‐state methodology showed that clofarabine exerts a strong antileukemic effect irrespective of MRD status and post‐remission treatment with alloSCT, which translates into improved current leukemia‐free survival (CLFS) as a novel composite endpoint for the group of patients randomized to clofarabine. With this type of additional analysis, we demonstrate that multi‐state models complement standard survival analysis methods and can be used to study the contributions of consecutive and competing events to standard and new composite survival outcomes.

## 2 METHODS

### 2.1 Study protocol and subjects

The HOVON102AML/SAKK30/09 study included patients with newly diagnosed AML and high‐risk myelodysplastic syndrome (refractory anemia with excess blasts with International Prognostic Scoring Scale ≥1.5), aged between 18 and 65 years. Patients were randomized between two cycles of standard remission‐induction chemotherapy with or without clofarabine. Here, we included all eligible patients randomized between the standard arm and the experimental arm with clofarabine 10 mg/m^2^. Patients achieving CR within two remission‐induction cycles received post‐remission treatment with either a third cycle of chemotherapy, or high‐dose chemotherapy followed by autologous stem cell transplantation or an alloSCT, as described previously.^10^

### 2.2 Definitions

The definition of CR was modified from the International Working Group Criteria.^10^, ^17^ MRD was assessed after two cycles of induction chemotherapy, or after the first cycle if no sample was available after the second cycle. MRD was assessed by flow cytometry and considered negative (MRD‐) if the leukemia‐associated immunophenotype was detected in less than 0.1% in the white blood cell compartment as validated previously.^18^ AlloSCT was considered as an event irrespective of timing of the transplant as long as the patient had not relapsed before alloSCT (in 1st remission). Relapse was defined according to the criteria described in the study protocol.^10^ Death from all causes was considered. All mortality occurring after relapse is denoted as relapse mortality (RM). All other deaths occurring before/without relapse, and including deaths due to lack of response/progressive disease after induction therapy, are denoted as NRM.

We defined the outcome measure of CLFS as being alive in CR before alloSCT (state “CR” meaning in CR after induction treatment followed by a post‐remission therapy other than alloSCT (if any)) or relapse‐free after alloSCT (state “AlloSCT” [adapted from the original definition by Klein et al.^19^]). In contrast to standard survival endpoints where the probability can only decrease over time due to failures, CLFS probability is 0 at time =0, and increases over time with entry events (CR or alloSCT) and decreases due to exit events (relapse or death).

### 2.3 Statistical analyses

**FIGURE 1:**
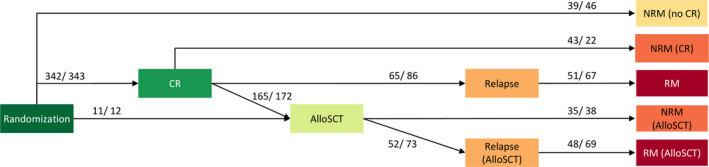
Multi‐state model: Each event of interest is represented as a separate state. All patients start in the state “Randomization” at time 0. The arrows depict all possible transitions a patient is at risk for. The preceding state is denoted in brackets. A patient remains in the current state until the next event occurs or censoring (at the end of follow‐up) occurs. “NRM” and “RM” states are absorbing, meaning that no further transitions are possible once a patient enters any of these states. Event counts per treatment arm “clofarabine/ standard” are listed for each transition. CLFS is defined as the sum of the probabilities of being in state “CR” or “AlloSCT” at a given point in time. AlloSCT, allogeneic stem cell transplantation; CR, complete remission; NRM, non‐relapse mortality; RM, relapse mortality (all mortality taking place after relapse).

Time was measured from randomization in all analyses, and patients were analyzed according to the treatment arm they were randomized to. For consistency with the primary publication,^10^ patients who were considered ineligible at hindsight were excluded from all analyses. In order to study the difference in relapse and death between the treatment arms before and after alloSCT (in 1st remission), we used the multi‐state model structure presented in Figure 1.^20^, ^21^ All patients start in the “Randomization” state. A patient remains in the current state until one of the modelled events CR, alloSCT (in 1st remission), relapse, or death occurs. We developed time in‐homogeneous Markov multi‐state models meaning that the hazard of transition from one state to another does not depend on the time spent in the current state, but only on the current state and the time since randomization. The effect of the treatment arm on the hazard of each transition is modelled using a semi‐parametric Cox proportional hazards model. Schoenfeld residuals were used to test for violations of the proportional hazards assumption. The relative differences between the treatment arms are expressed in terms of hazard ratios (HR).

Subgroup analysis was performed by separately estimating the same semi‐parametric multi‐state model in each of the four subgroups defined by European LeukemiaNet (ELN) 2010 risk classification.^22^

For the purpose of studying the role of MRD status, we built a separate model where the “CR” state was split into three MRD states: MRD‐, MRD+, and MRD‐unknown (MRDunk).

Additional specification of the statistical analyses is provided in the supplement.

All analyses were performed in R, version 3.6.0, using the packages “survival” and “mstate”.^21^, ^23^

## 3 RESULTS

### 3.1 Patient cohort

Between 25 February 2010 and 28 September 2013, 412 patients were randomized to the standard arm and 413 to the clofarabine arm. In total 30 patients (10 in the standard arm and 20 in the experimental arm) were considered ineligible at hindsight and were excluded, leaving 795 patients for the analyses. Study population, treatment, and standard clinical outcomes were previously reported.^10^

At data lock on 24 May 2019 the median follow‐up was 72 months (range 10–108 months), 18 patients were lost to follow‐up, and 216 patients in the clofarabine arm and 242 patients in the standard arm had died. The CR rates within 6 months did not differ between the treatment arms (87% in the clofarabine arm vs. 85% in the standard arm). The median time to post‐remission therapy with alloSCT was 3.9 months since randomization (interquartile range 3.2–4.8 months). A total of 176 (45%) patients in the clofarabine arm received alloSCT versus 184 (46%) patients in the standard arm.

### 3.2 Multi‐state model

**FIGURE 2:**
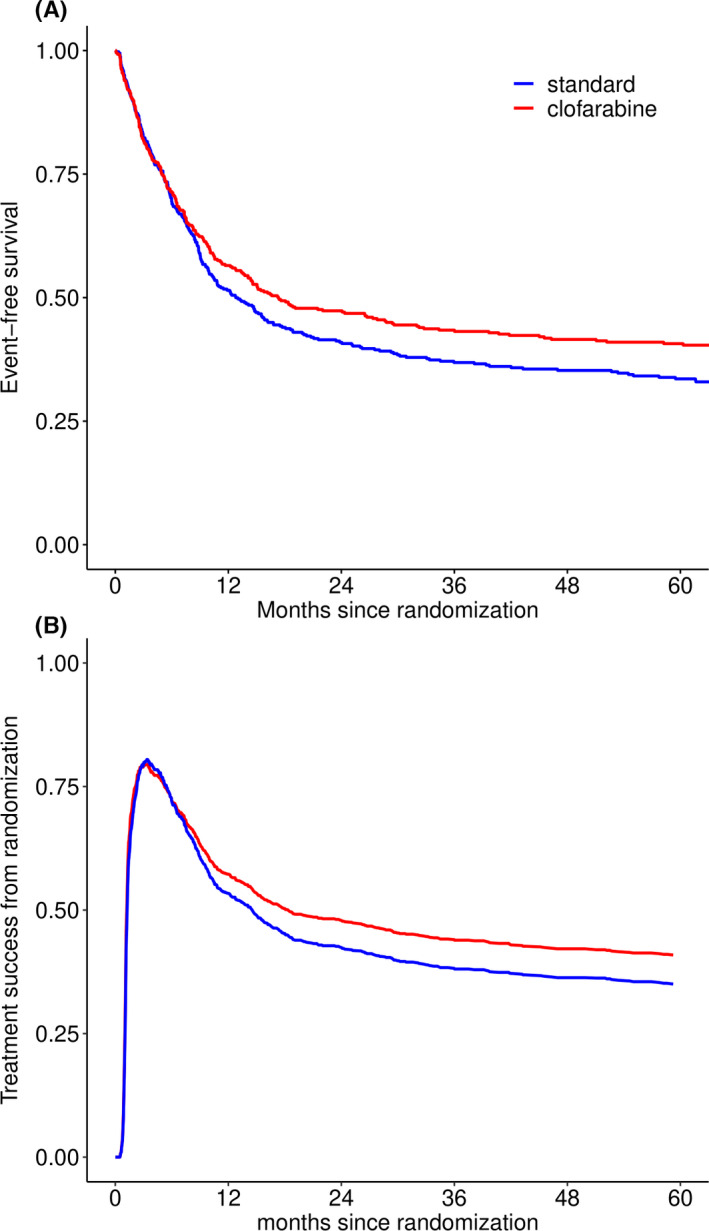
Panel A: Event‐free survival since randomization where events are no CR at the end of induction treatment, relapse, or death. Updated result from Löwenberg et al. (Blood 2017) with median follow‐up of patients still alive of 72 months (range 10–108 months). Panel B: Current leukemia‐free survival: Probability of current leukemia‐free survival over time per treatment arm, where CLFS is defined as the sum of the probabilities of being in state “CR” or “AlloSCT” at a given point in time, based on the multi‐state model in Figure 1. AlloSCT, allogeneic stem cell transplantation, CR, complete remission.

Longer follow‐up data yield similar results for the primary study endpoint as reported in the original publication^10^ with no sufficient evidence for an OS (HR 0.91, 95% confidence interval [CI] 0.76–1.10, *p* = 0.34, supplemental Figure S1), or an EFS (HR 0.85, 95% CI 0.72–1.02, *p* = 0.08, Figure 2A) benefit in patients treated with clofarabine. Cumulative incidence curves of relapse and NRM are presented in the supplemental Figure S2. We built the multi‐state model in Figure 1, which distinguishes between relapse in patients who did and did not receive alloSCT (in 1st remission), to further investigate the previously reported reduced hazard of relapse in the clofarabine arm.^10^ We found that clofarabine compared to standard treatment is associated with lower risk of relapse after achievement of CR and possible post‐remission treatment: HR 0.67 (95% CI 0.47–0.95, *p* = 0.02) for recipients of alloSCT and HR 0.78 (95% CI 0.57–1.08, *p* = 0.14) for patients not receiving alloSCT. Hazard ratios of the clofarabine arm versus standard arm for all transitions are presented in supplemental Table S1. There was no evidence for violations of the proportional hazards assumption.

**FIGURE 3:**
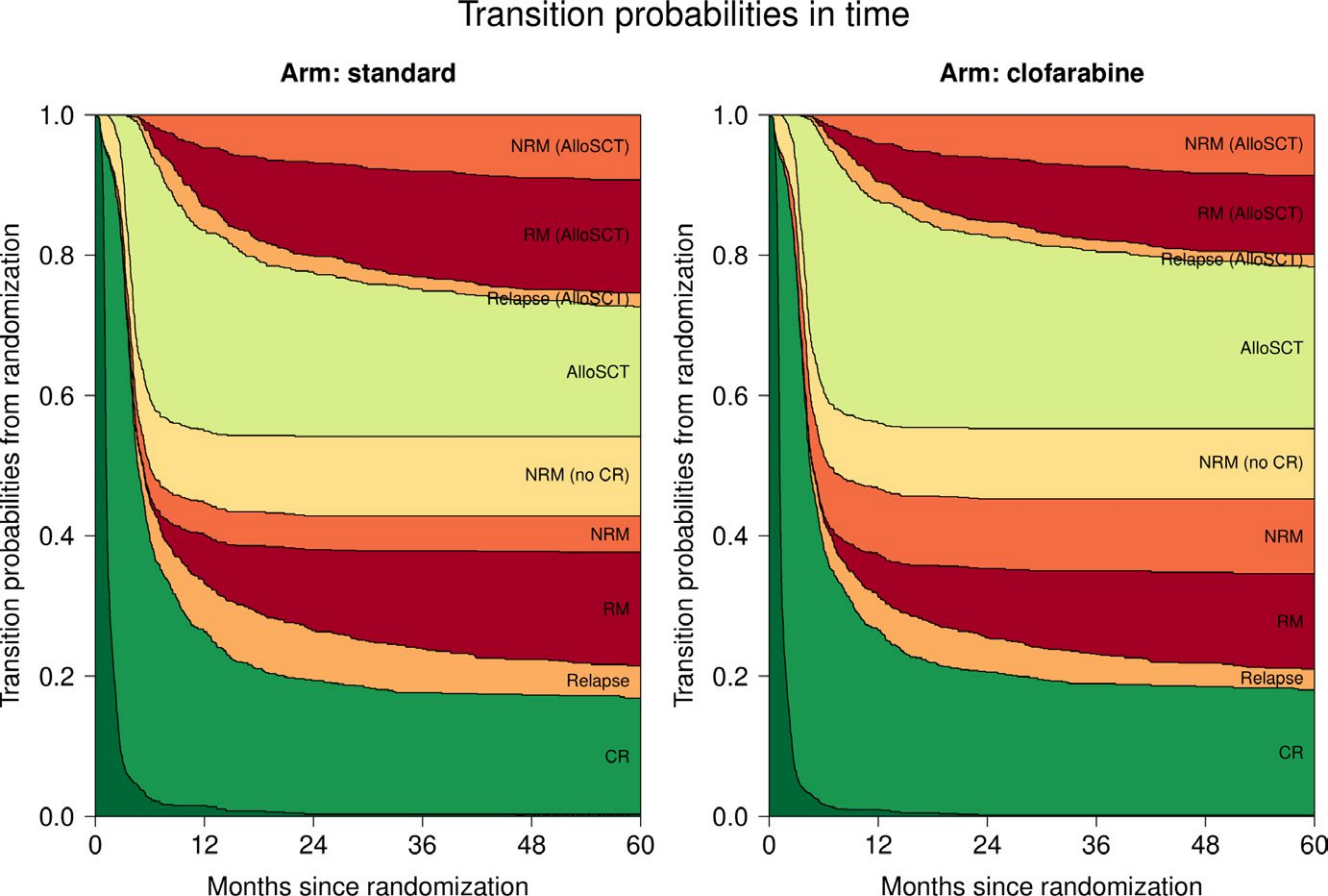
Transition probabilities to all states from randomization: Transition probabilities derived from the multi‐state model (see Figure 1). At each point in time, the distance between two adjacent curves represents the probability of being in the corresponding state, conditional on being in state “Randomization” at time 0. The probability of being in an intermediate state can both increase and decrease over time, while the probability of absorbing (death) states can only increase over time. Transition probabilities (plus 95% CI) at 24 and 60 months since randomization are listed in supplemental Table S3. AlloSCT, allogeneic stem cell transplantation, CR, complete remission, NRM, non‐relapse mortality, RM, relapse mortality (all mortality taking place after relapse).

The multi‐state model distinguishes between NRM before achievement of CR (state “NRM (no CR)”), after CR (state “NRM (CR)”), and after post‐remission treatment with alloSCT (state “NRM (AlloSCT)”). We compared the relative difference in the incidence of these three types of NRM between the treatment arms (Table S1), and found that the main contributor to the previously reported overall higher incidence of NRM in the clofarabine arm versus the standard arm is NRM before post‐remission treatment with alloSCT (state “NRM (CR)” in Figure 3, HR 2.02, 95% CI 1.21–3.37, *p* = 0.01).^10^ The causes of death for patients in each of the three NRM states are summarized in Table S2.

### 3.3 Current leukemia‐free survival

In order to assess the net benefit of treatment with clofarabine, we employed the outcome measure of CLFS, which is defined as the sum of the probabilities of being in either the state “CR” or “AlloSCT”, in other words, being alive in CR before alloSCT (or possibly having undergone another post‐remission therapy), or alive and relapse‐free after alloSCT (transplant in 1st remission). The probability of CLFS over time is shown in Figure 2B. CLFS increases within the first 4 months from randomization as patients achieve CR and receive post‐remission treatment with alloSCT. The CLFS curves of the two treatment arms start to diverge after 8 months since randomization. The probability of being in one of the CLFS states decreases to 57% and 53% after 1 year, and reaches 41% and 35% after 5 years in the clofarabine and the standard arm, respectively.

### 3.4 Multi‐state model with MRD status

**FIGURE 4:**
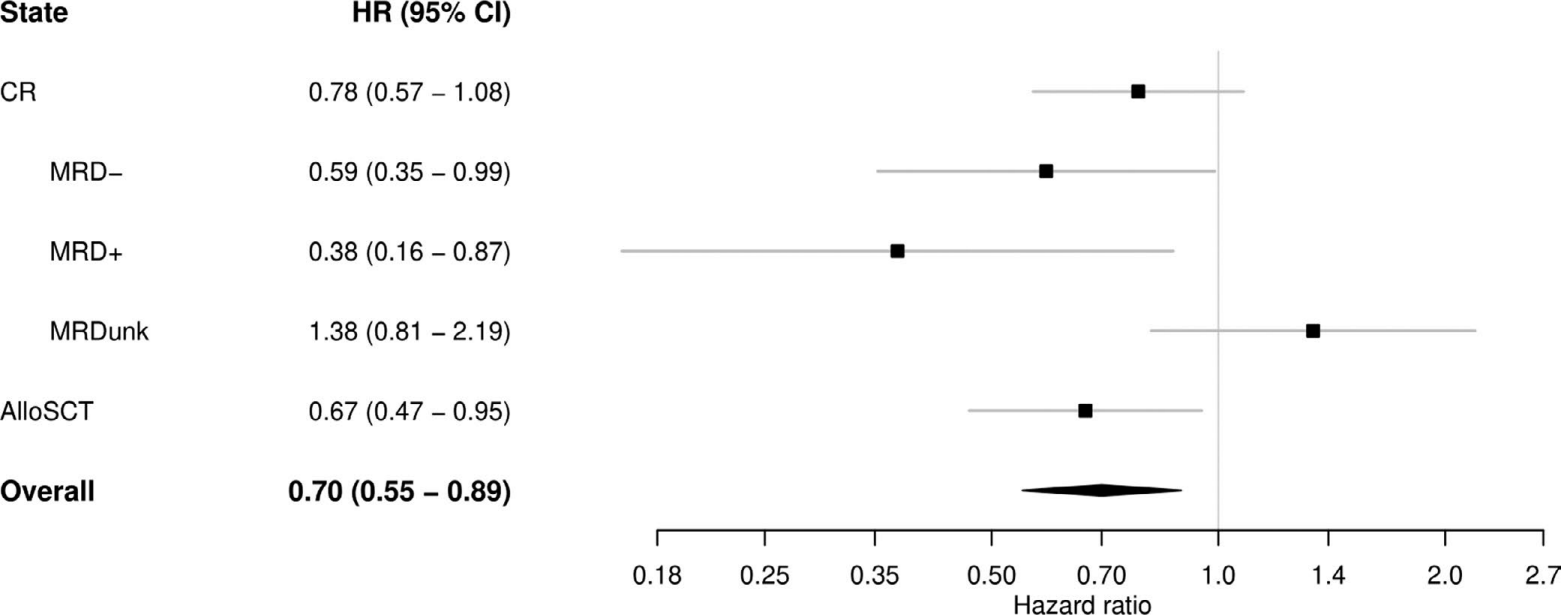
Forest plot: Estimated hazard ratios of the clofarabine arm versus standard arm for the transitions to relapse starting from different states (plus 95% confidence intervals). “CR” for patients currently in CR without having experienced alloSCT (possibly having undergone another post‐remission treatment), and “AlloSCT” for patients receiving alloSCT. In a separate model we split the patients in CR (without/before having experienced alloSCT) according to their MRD status. The overall estimate is the hazard ratio of the transition from CR to relapse, ignoring alloSCT. A hazard ratio of less than 1 (to the left of the vertical line) indicates lower risk of relapse for the clofarabine arm compared to the standard arm. AlloSCT, allogeneic stem cell transplantation, CI, confidence interval, CR, complete remission, HR, hazard ratio, MRD‐, measurable residual disease negativity, MRD+, measurable residual disease positivity, MRDunk, unknown measurable residual disease status.

It has previously been shown that achievement of MRD‐ is associated with a better prognosis.^15^, ^24^, ^25^, ^26^ In this study we assessed the effect of treatment with clofarabine on relapse based on MRD status. We split the “CR” state into three states: “MRD‐", “MRD+”, and “MRDunk”. MRD status determined by flow cytometry was available in 53% of the CR patients (54% in the clofarabine arm and 51% in the standard). We observed 278 patients achieving MRD‐, 150 (44% of CR patients) and 128 (37%) in the clofarabine and standard arm, respectively (HR 1.12, 95% CI 0.88–1.42, *p* = 0.34, Figure S3). The forest plot in Figure 4 presents the estimated hazard ratios of the clofarabine arm versus standard arm for the transition from each MRD state to relapse (i.e., during the time period between achieving CR and possible post‐remission treatment with alloSCT). MRD‐ patients treated with clofarabine have a lower hazard of relapse than the patients in the standard arm (HR 0.59, 95% CI 0.35–0. 99, *p* = 0.05). A similar decreased hazard of relapse by clofarabine was observed in MRD+ patients, which might indicate that clofarabine induces deeper remission not captured by the dichotomization of MRD status.

No statistically significant difference in the hazard of relapse was found between the standard and the clofarabine arm in patients with unknown MRD status. In addition, no apparent differences were present in baseline characteristics between the treatment arms in the MRDunk subgroup. We hypothesize that an imbalance between the treatment arms in distribution of MRD‐ and MRD+ status among MRDunk patients might drive this observation.

We further investigated the hypothesis that clofarabine might induce deeper remission not captured by the dichotomization of MRD status. The median percentage of the leukemia‐associated immunophenotype in the white blood cell compartment was lower in the clofarabine arm 0.01% (mean 0.016%, IQR 0.00–0.02%) compared with 0.02% (mean 0.023%, IQR 0.01–0.03%) in the standard arm in MRD‐ patients; Wilcoxon p‐value 0.03. Among MRD+ patients no significant difference was found in the levels of leukemia‐associated immunophenotype in the clofarabine versus the standard arm (median 0.44% vs. 0.52%, respectively, *p* = 0.68).

### 3.5 Transition probabilities

A favorable feature of multi‐state models is the possibility to integrate the underlying hazards of events with the estimated relative effects of the treatment arms, and thus derive probabilities of being in a certain state at different points in time (Figure 3). The total probability of relapse after each intermediate event is equal to the sum of the respective “Relapse” and “Relapse mortality” states. Comparing the probabilities of being in these states between the treatment arms shows that the relative reduction of relapse in patients with and without post‐remission treatment with alloSCT translates to an absolute benefit in terms of relapse when randomized into the clofarabine arm. Transition probabilities (plus 95% CI) at 24 and 60 months since randomization are listed in Table S3.

**FIGURE 5:**
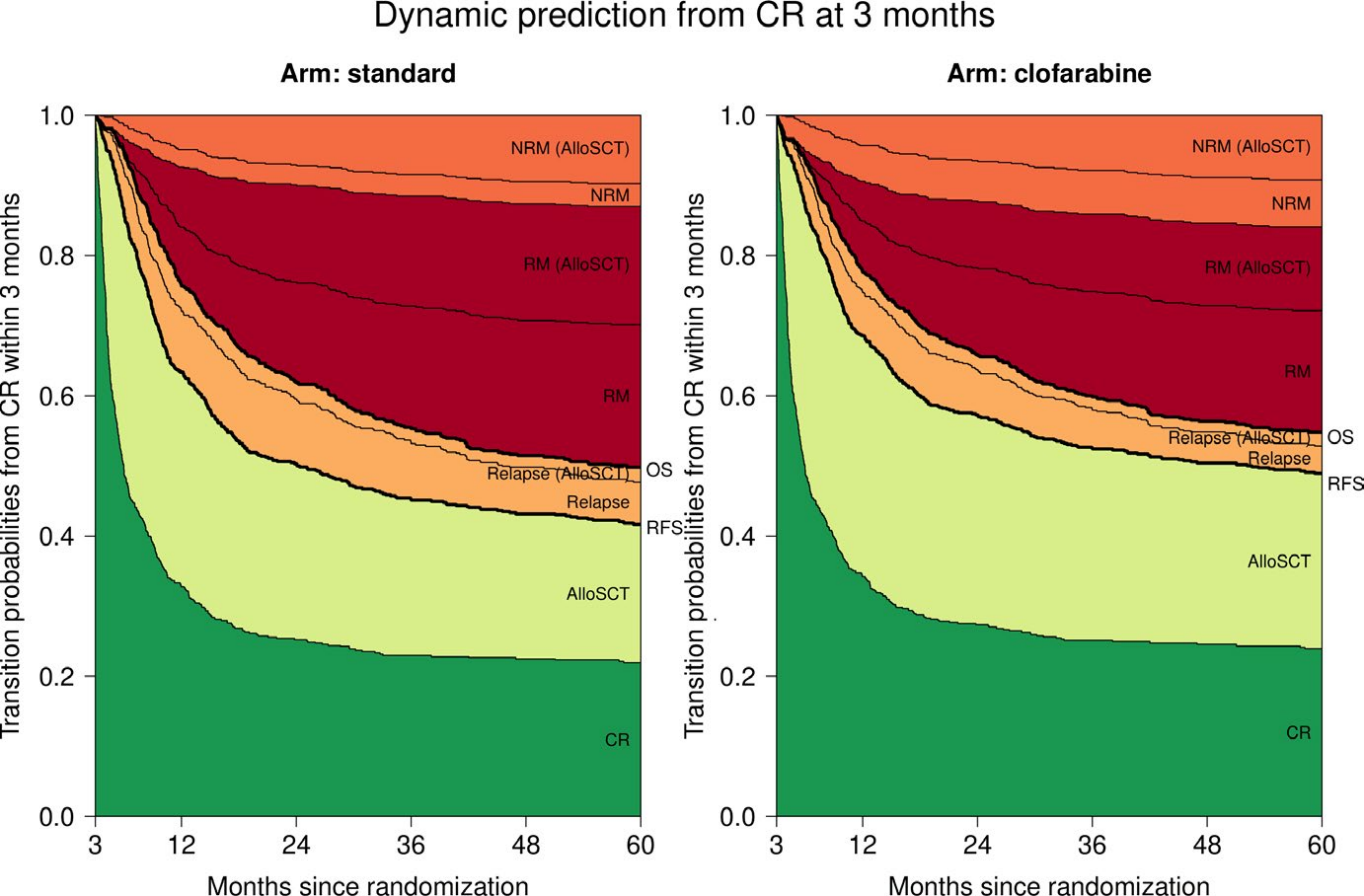
Transition probabilities to all states from the CR state at 3 months since randomization (see multi‐state model in Figure 1). At each point in time, the distance between two adjacent curves represents the probability of being in the corresponding state for patients in state “CR” (see Figure 1) at 3 months since randomization. Transition probabilities are easily combined to construct standard survival outcomes like RFS from CR and OS. Transition probabilities (plus 95% CI) at 24 and 60 months since randomization are listed in supplemental Table S4. AlloSCT: allogeneic stem cell transplantation, CR, complete response; NRM, non‐relapse mortality, OS, overall survival, RFS, relapse‐free survival from CR, RM, relapse mortality (all mortality taking place after relapse), TP, transition probability.

Multi‐state models also enable to calculate the transition probabilities from any state at later time points. Figure 5 presents the transition probabilities conditional on being in the “CR” state at 3 months since randomization (exact estimates at 24 and 60 months are listed in Table S4). The probability of undergoing alloSCT is comparable between the treatment arms (sum of the transition probabilities to the states “AlloSCT”, “Relapse (AlloSCT)”, “RM (AlloSCT)”, and “NRM (AlloSCT)”), just as the probability of being alive after relapse in patients undergoing alloSCT as post‐remission treatment (“Relapse (AlloSCT)”). Compared to the standard arm, less patients are expected to relapse and subsequently die if treated with clofarabine irrespective of the type of post‐remission therapy (transition probabilities to the states “RM” and “RM (AlloSCT)” are lower for the clofarabine arm).

Like CLFS in Figure 2B, we can sum the transition probabilities of being in the “CR” and “AlloSCT” states to the relapse‐free survival (RFS) curves from CR achieved within 3 months, or sum the transition probabilities of being in the “CR”, “AlloSCT”, and both (alive after) relapse states to OS curves (emphasized in Figure 5). Consequently, multi‐state models allow us to study the composition of these endpoints in terms of intermediate events and competing risks.

### 3.6 Subgroup analysis by ELN 2010

As in the previous publication of this trial,^10^ we investigated possible subgroup effects based on ELN 2010 risk classification. For this purpose, we estimated the main multi‐state model in each subgroup defined by ELN risk group (Table S5). In none of the subgroups there were evidence for violations of the proportional hazards assumption.

**FIGURE 6:**
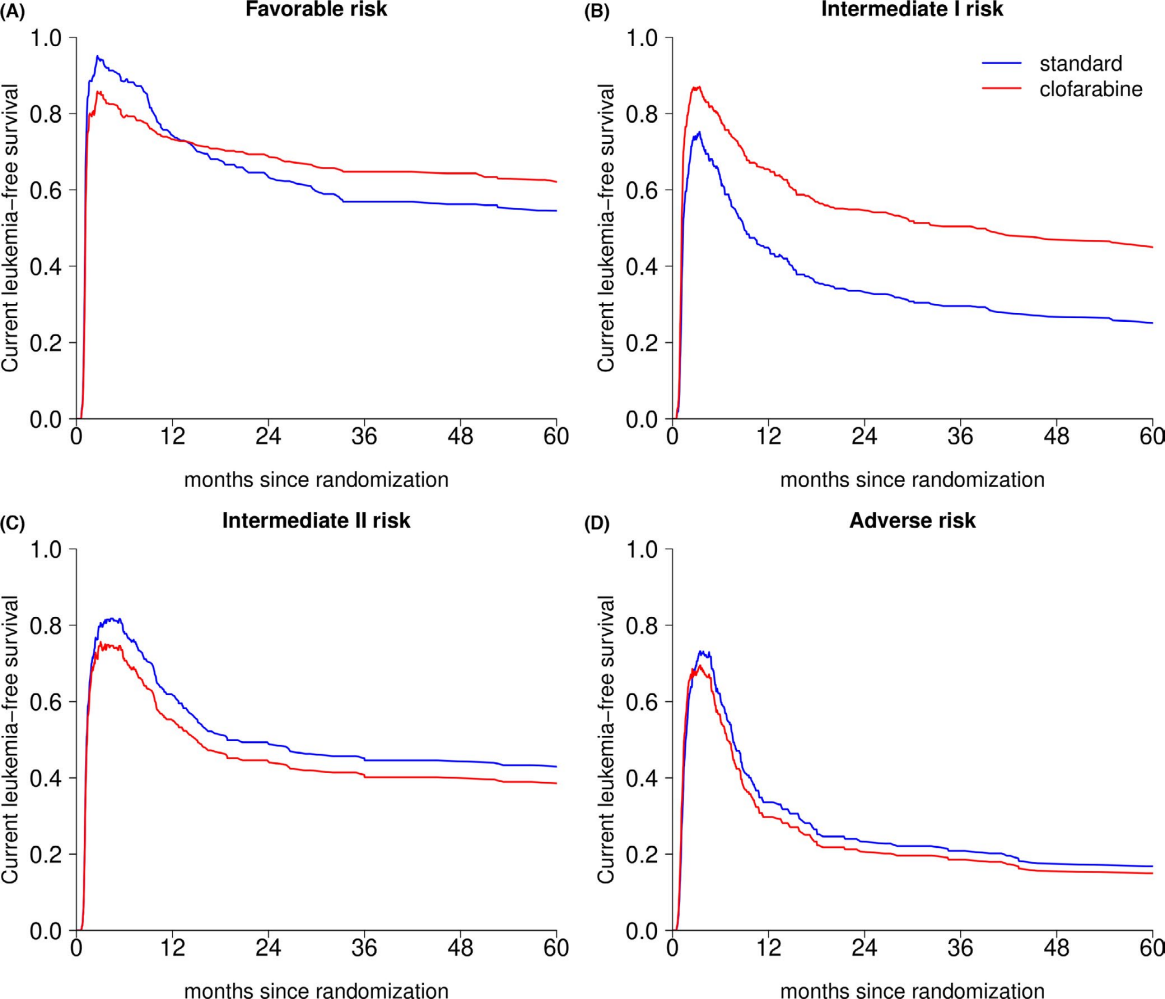
Current leukemia‐free survival by treatment arm in each of the ELN risk groups: current leukemia‐free survival is defined as the sum of the probabilities of being in state “CR” or “AlloSCT” at a given point in time (based on the multi‐state model in Figure 1). Each panel presents one ELN risk group: panel A ‐ Favorable risk, panel B ‐ Intermediate I, panel C ‐ Intermediate II, and panel D ‐ Adverse risk group. AlloSCT, allogeneic stem cell transplantation; CR, complete remission, ELN, European Leukemia Net.

**FIGURE 7:**
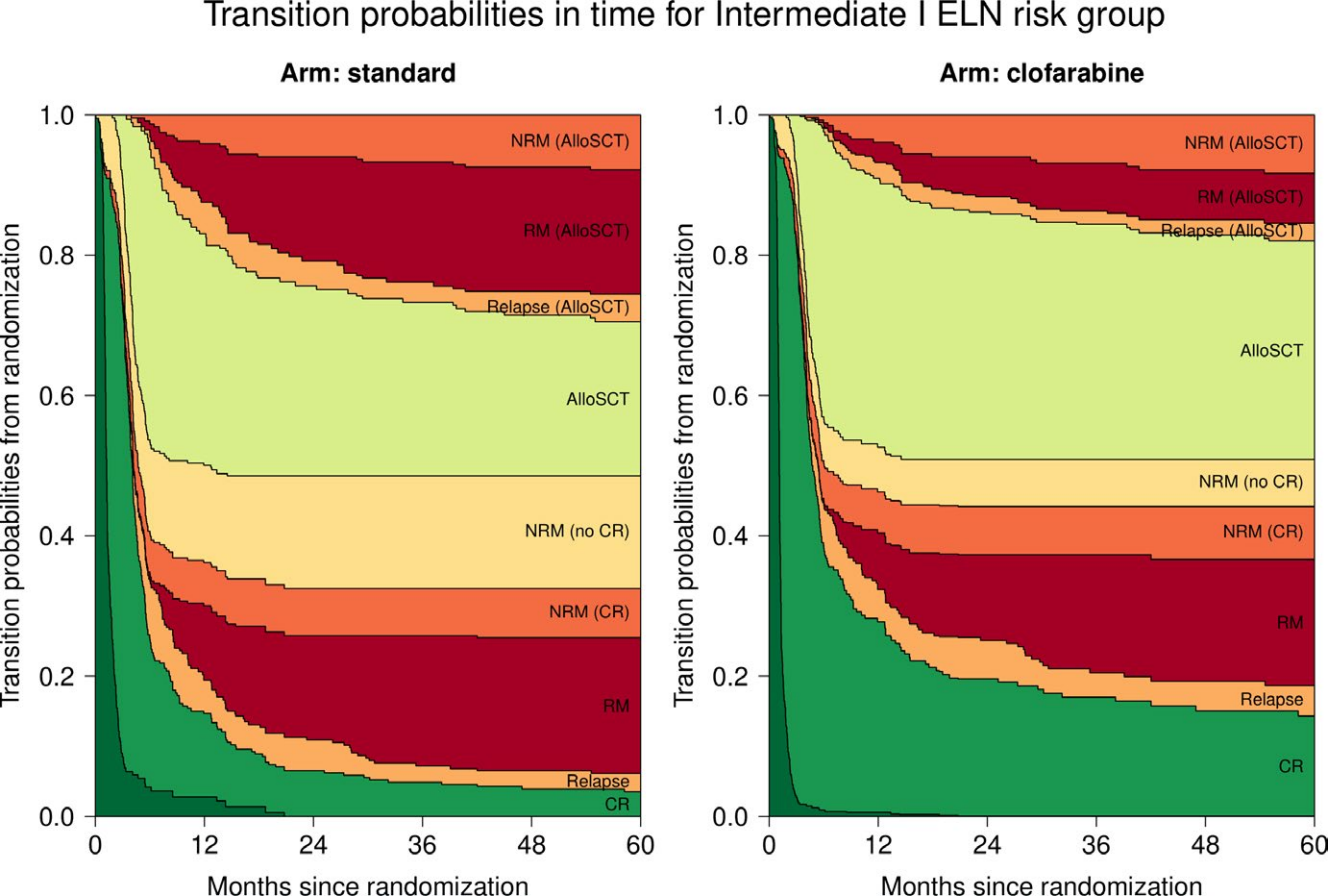
Transition probabilities to all states from randomization for the Intermediate I European LeukemiaNet (ELN) risk group: Semi‐parametric estimates of the transition probabilities to all states from randomization for the Intermediate I ELN risk group by treatment arm (based on the multi‐state model in Figure 1). The transition probabilities for the rest of the risk groups are presented in supplemental Figure S4. AlloSCT, allogeneic stem cell transplantation; CR, complete remission; ELN, European LeukemiaNet; NRM, non‐relapse mortality; RM, relapse mortality (all mortality taking place after relapse).

The difference in CLFS between the treatment arms is most pronounced in the intermediate I risk group, where CLFS is higher in the clofarabine arm (Figure 6). Similar results with respect to EFS were presented in the primary study publication.^10^ However, here we extend our understanding of these results by investigating the occurrence of relapse and death before and after post‐remission treatment with alloSCT. For the intermediate I risk group (Figure 7), the difference in CLFS between the treatment arms in the first 6 months since randomization is primarily driven by the higher probability of NRM for patients treated in the standard arm who do not achieve CR (6.3% vs. 13.0% transition probability to state “NRM (no CR)” at 6 months for the clofarabine vs. standard arm, respectively). This difference increases beyond 6 months due to the higher probability of relapse and relapse followed by death for patients in the standard arm undergoing alloSCT (5.7% vs. 14.8% transition probability to state “RM (AlloSCT)” at 24 months, Table S6). The transition probabilities for the rest of the risk groups are presented in Figure S4.

## 4 DISCUSSION

Clofarabine is an active antileukemic drug in AML which increases response rates,^3^, ^4^, ^5^, ^6^, ^7^, ^8^, ^9^, ^10^, ^11^, ^12^, ^13^ and has also been associated with promising outcomes when used as salvage treatment for patients with relapsed or refractory AML patients as bridge to alloSCT.^27^, ^28^ However, the beneficial effect of clofarabine was earlier suggested to be restricted to subgroups of patients. The analysis of clinical trials for patients with AML is complex, since these patients may experience different beneficial and detrimental clinical events during induction, post‐remission treatment, and follow‐up. In addition, post‐remission treatment in AML varies according to risk category, which may further complicate the interpretation of an ITT analysis.^9^, ^10^, ^15^ By using multi‐state methodology, we evaluated the effect of the intermediate events CR, MRD status, and alloSCT (in 1st remission) on the hazard of relapse and death. Compared to the standard arm, the hazard of relapse was significantly lower in the clofarabine arm, which effect was observed among patients who received post‐remission treatment with alloSCT as well as in CR patients who were not allografted. Furthermore, clofarabine reduced the hazard of relapse in both MRD‐ and in MRD+ patients, although missing data (46% of patients) preclude strong conclusions. NRM in CR patients not receiving alloSCT was higher in the clofarabine arm compared to the standard arm, indicating that some of the favorable effects of clofarabine on relapse may be compromised by toxicity‐related mortality. In addition, we introduced the composite survival endpoint CLFS, and showed an overall benefit of randomization to clofarabine in terms of CLFS.

Multi‐state models are increasingly being applied in cancer research, particularly in the context of intermediate events.^29^, ^30^, ^31^, ^32^, ^33^, ^34^ Despite the encouraging publication of Le‐Rademacher et al,^35^ these models have rarely been applied for re‐analysis of RCTs.^35^, ^36^ Multi‐state models have a number of advantages over other existing methods, including censoring at intermediate events, time‐dependent Cox regression, and landmarking. First, the method of censoring survival at intermediate events like time‐dependent treatments has been regularly applied to adjust survival outcomes for the effect of intermediate events.^10^, ^37^, ^38^, ^39^, ^40^ However, when OS is censored at the time of alloSCT, informative censoring is being introduced leading to biased estimates of treatment effects. On the one hand, patients who undergo alloSCT have a different risk profile than the patients who are not allografted, and on the other hand, alloSCT strongly influences prognosis, both leading to different outcomes after the censoring time for the censored and non‐censored patients.

Second, landmarking is inefficient for statistical comparisons, since it excludes all patients who reach the endpoint before the landmark, arbitrary due to the subjective choice of the landmark time, and conservative compared to time‐dependent Cox regression.^41^ Post‐remission treatment can also be modelled as a time‐dependent covariate in a Cox proportional hazards model.^9^, ^42^, ^43^ This approach, however, assumes a time‐independent constant ratio of the hazards, with and without the intermediate event. That assumption is not valid when the intermediate event is alloSCT due to the initial high risk of NRM and relapse, followed by a decrease in the hazard. Yet, this method is more flexible and accurate than the preceding ones, and it gives a valid averaged over time effect estimate.

Multi‐state methods overcome these limitations and present new possibilities in the form of dynamic prediction and novel composite endpoints.^19^, ^29^, ^36^, ^44^, ^45^ However, when applied to randomized controlled trials, multi‐state models should be interpreted with caution as they introduce selection on the basis of the observed intermediate events. While comparing the outcomes between the treatment arms after the intermediate event, one should keep in mind that at this point the arms are no longer balanced with respect to risk factors as we implicitly condition on experiencing the intermediate event.

Our study has a number of limitations. First, we have developed Markov models in which the transition probabilities do not depend on the time spent in the current state, but only on the current state itself and the time since randomization. In the context of alloSCT this assumption may be questioned. AlloSCT is associated with an increased risk of NRM, particularly in the early phase after alloSCT. In this case, modelling the effect of time since alloSCT may be of interest for some transitions. Second, we aimed to study whether clofarabine reduces the hazard of relapse by inducing deeper remission as measured by MRD‐. Although the data suggest supporting this hypothesis, our findings are compromised by missing MRD status in 46% of the CR patients.

In conclusion, we have used multi‐state models to further elucidate the previously reported reduced hazard of relapse in the clofarabine arm of the HOVON102AML/SAKK30/09 prospective, randomized, controlled, phase III trial. We found a lower hazard of relapse in the clofarabine arm compared to the standard arm even after post‐remission treatment with alloSCT. Altogether, these results suggest that clofarabine provides an active antileukemic effect when added to induction treatment for AML patients aged 65 and younger. However, when taking the higher probability of NRM into account, the net difference between the treatment arms, expressed by CLFS, was reduced. The methods presented here generated additional insights into the effects of a series of treatments by studying the sequence of various events which take place after randomization in a RCT.

## CONFLICT OF INTEREST

The authors declare no conflicts of interest.

## AUTHOR CONTRIBUTION

Katerina Bakunina, Hein Putter, Jurjen Versluis, Jan J. Cornelissen, Liesbeth C. de Wreede. participated in the data analyses and drafted the manuscript. All other co‐authors contributed to the final version of the manuscript.

## ETHICS APPROVAL

The HOVON 102 RCT was approved by all respective ethics committees, however the novel research presented is not subject to ethical approval.

## Supporting information

Supplementary Material

## Data Availability

Data sharing not applicable – no new data generated.
