# Supplementary material for: The added value of multi‐state modelling in a randomized controlled trial: The HOVON 102 study re‐analyzed
Source: Cancer Med. 2021 Dec 24;11(3):630–40. doi: 10.1002/cam4.4392 (PMC8817075; doi:10.1002/cam4.4392)

The added value of multi-state modelling in a randomized controlled trial: the HOVON 102 study re-analyzed

Katerina Bakunina${}^{1}$, Hein Putter${}^{2}$, Jurjen Versluis${}^{3}$, Eva A. S. Koster${}^{4}$, B. van der Holt${}^{1}$, Markus G. Manz${}^{5}$, Dimitri A. Breems${}^{6}$, Bjorn T. Gjertsen${}^{7}$, Jacqueline Cloos${}^{8}$, Peter J. M. Valk${}^{3}$, Jakob Passweg${}^{9}$, Thomas Pabst${}^{10}$, Gert J. Ossenkoppele${}^{8}$, Bob Löwenberg${}^{3}$, Jan J. Cornelissen${}^{3}$, Liesbeth C. de Wreede${}^{2}$

# Supplemental material

**Statistical analyses**

Subgroup analysis was performed by separately estimating the same semi-parametric multi-state model in each of the four subgroups defined by European LeukemiaNet (ELN) 2010 risk classification.^1^ We chose this approach since the arms were well balanced within the risk groups, we could not justify the assumption of proportionality of the hazards in the different risk groups, and an insufficient number of events was observed to estimate all risk group and treatment arm interaction effects.

For the purpose of studying the role of MRD status, we built a separate model where the “CR” state was split into three MRD states: MRD-, MRD+, and MRD-unknown (MRDunk). Date of MRD- is defined as the date of the sample after two remission-induction cycles if it fulfilled MRD- criteria. Date of MRD+ is defined as the date of the sample after two remission-induction cycles if it did not fulfill MRD- criteria. For patients whose MRD status after two cycles was unknown, the sample date and status after the first remission-induction cycle was used, if available. Date of MRDunk is defined as the date of first response evaluation fulfilling CR criteria, for patients with unknown MRD status after one and two remission-induction cycles.

The multi-state modelling framework allows to integrate the underlying hazards of events with the estimated relative effects of the treatment arms, and thus derive absolute measures of risk called transition probabilities. Analogous to survival probabilities, transition probabilities are defined as the probability of being in a certain state at a point in time, conditional on being in (possibly) another state at the time the prediction is made. By default, predictions are made from time 0 (randomization), when all patients start in the initial state, but the time of the prediction can be varied just as the state from which the prediction is made. For example, when a patient comes for a follow-up visit, the prognosis can be updated using the information that the patient is in a given state at the visit time, which is referred to as dynamic prediction.^2^ Estimated transition probabilities can also be combined into useful measures like CLFS,^3,4^ and treatment success.^5,6^

CLFS over time was calculated by taking the sum of the transition probabilities of being in state “CR” and “AlloSCT” over time, conditional on being in the “Randomization” state at time 0.

**References**

1 Döhner H, Estey EH, et al. Diagnosis and management of acute myeloid leukemia in adults: recommendations from an international expert panel, on behalf of the European LeukemiaNet. *Blood*. 2010;115(3):453-474.

2 van Houwelingen H, Putter H. Dynamic prediction in clinical survival analysis. (CRC Press, Boca Raton, 2011).

3 Klein JP, Szydlo RM, Craddock C, Goldman JM. Estimation of current leukaemia‐free survival following donor lymphocyte infusion therapy for patients with leukaemia who relapse after allografting: application of a multistate model. *Statistics in medicine*. 2000;19(21):3005-3016.

4 Liu L, Logan B, Klein JP. Inference for current leukemia free survival. *Lifetime data analysis*. 2008;14(4):432-446.

5 Eefting M, de Wreede LC, et al. Multi-state analysis illustrates treatment success after stem cell transplantation for acute myeloid leukemia followed by donor lymphocyte infusion. *Haematologica*. 2016;101(4):506-514.

6 Bluhmki T, Schmoor C, Finke J, Schumacher M, Socié G, Beyersmann J. Relapse-and Immunosuppression-Free Survival after Hematopoietic Stem Cell Transplantation: How Can We Assess Treatment Success for Complex Time-to-Event Endpoints?. *Biology of Blood and Marrow Transplantation*. 2020;26(5):992-997.

**Results**

**Table S1**: Results of the multi-state model. Hazard ratios of the clofarabine arm versus standard arm for all transitions, based on transition-specific proportional hazards models (plus 95% CI).
AlloSCT: allogeneic stem cell transplantation, CI: confidence interval, CR: complete remission, HR: hazard ratio, NRM: non-relapse mortality, RM: relapse mortality (all mortality taking place after relapse).

| **Transition** | **HR** | **95% CI** | **p-value** |
| --- | --- | --- | --- |
| Randomization -> CR | 1.12 | (0.97-1.31) | 0.13 |
| Randomization ->AlloSCT | 1.12 | (0.49-2.55) | 0.79 |
| Randomization -> NRM (no CR) | 1.03 | (0.67-1.58) | 0.89 |
| CR -> AlloSCT | 1.00 | (0.80-1.23) | 0.96 |

| **Transition** | **HR** | **95% CI** | **p-value** | **Transition** | **HR** | **95% CI** | **p-value** |
| --- | --- | --- | --- | --- | --- | --- | --- |
| CR -> Relapse | 0.78 | (0.57-1.08) | 0.14 | AlloSCT -> Relapse (AlloSCT) | 0.67 | (0.47-0.95) | 0.02 |
| CR-> NRM (CR) | 2.02 | (1.21-3.37) | 0.01 | AlloSCT-> NRM (AlloSCT) | 0.84 | (0.53-1.32) | 0.45 |
| Relapse -> RM | 1.20 | (0.83-1.73) | 0.34 | Relapse (AlloSCT) -> RM (AlloSCT) | 0.89 | (0.61-1.30) | 0.54 |

***Table S2****:* *Cause of death for patients in each of the three NRM states
NRM: non-relapse mortality (defined as any death before/without disease relapse, including progression, or death due to complications before achieving CR)*

| **State** | **Cause of death** | **Standard** | **Clofarabine** |
| --- | --- | --- | --- |
| NRM (no CR) | Other infection | 14 (30%) | 14 (36%) |
|  | AML | 17 (37%) | 5 (13%) |
|  | Pneumonitis | 1 (2%) | 8 (21%) |
|  | Multi organ failure | 4 (9%) | 4 (10%) |
|  | Other | 4 (9%) | 2 (5%) |
|  | Hemorrhage | 2 (4%) | 3 (8%) |
|  | Respiratory failure | 1 (2%) | 2 (5%) |
|  | Renal failure | 2 (4%) | 0 (0%) |
|  | Cerebral edema | 0 (0%) | 1 (3%) |
|  | Unknown | 1 (2%) | 0 (0%) |
|  | ***Total*** | ***46 (100%)*** | ***39 (100%)*** |
| NRM (CR) | Other infection | 7 (32%) | 15 (35%) |
|  | Hemorrhage | 1 (5%) | 8 (19%) |
|  | Multi organ failure | 3 (14%) | 6 (14%) |
|  | Pneumonitis | 3 (14%) | 5 (12%) |
|  | Cardiac events | 4 (18 %) | 1 (2%) |
|  | Other | 2 (9%) | 2 (5%) |
|  | Secondary malignancy | 1 (5%) | 2 (5%) |
|  | Respiratory failure | 0 (0%) | 3 (7%) |
|  | Unknown | 1 (5%) | 1 (2%) |
|  | ***Total*** | ***22 (100%)*** | ***43 (100%)*** |
| NRM (AlloSCT) | GvHD | 11 (29%) | 13 (37%) |
|  | Other infection | 10 (26%) | 14 (40%) |
|  | Pneumonitis | 6 (16%) | 4 (11%) |
|  | Other | 4 (11%) | 1 (3%) |
|  | Hemorrhage | 4 (11%) | 0 (0%) |
|  | Secondary malignancy | 3 (8%) | 1 (3%) |
|  | Multi organ failure | 0 (0%) | 2 (6%) |
|  | ***Total*** | ***38 (100%)*** | ***35 (100%)*** |

**Table S3**: Transition probabilities from randomization. Transition probabilities (plus 95% CI) at 24 and 60 months since randomization per treatment arm.
AlloSCT: allogeneic stem cell transplantation, CI: confidence interval, CR: complete remission, HR: hazard ratio, NRM: non-relapse mortality, RM: relapse mortality (all mortality taking place after relapse), TP: transition probability.

|  |  | **Standard arm** | | **Clofarabine arm** | |
| --- | --- | --- | --- | --- | --- |
| **Time** | **State** | **TP** | **95% CI** | **TP** | **95% CI** |
| 24 months | Randomization | 0.00 | 0.00 - 0.00 | 0.00 | 0.00 - 0.01 |
|  | CR | 0.19 | 0.15 - 0.23 | 0.20 | 0.17 - 0.24 |
|  | AlloSCT | 0.23 | 0.19 - 0.27 | 0.28 | 0.23 - 0.32 |
|  | Relapse | 0.07 | 0.05 - 0.10 | 0.05 | 0.03 - 0.07 |
|  | Relapse (AlloSCT) | 0.02 | 0.01 - 0.04 | 0.02 | 0.00 - 0.04 |
|  | NRM (no CR) | 0.11 | 0.09 - 0.14 | 0.10 | 0.08 - 0.12 |
|  | NRM (CR) | 0.05 | 0.03 - 0.07 | 0.10 | 0.07 - 0.13 |
|  | NRM (AlloSCT) | 0.07 | 0.04 - 0.09 | 0.06 | 0.04 - 0.08 |
|  | RM | 0.12 | 0.09 - 0.15 | 0.10 | 0.07 - 0.13 |
|  | RM (AlloSCT) | 0.13 | 0.10 - 0.16 | 0.09 | 0.06 - 0.12 |
| 60 months | Randomization | 0.00 | 0.00 - 0.00 | 0.00 | 0.00 - 0.01 |
|  | CR | 0.17 | 0.13 - 0.20 | 0.18 | 0.14 - 0.22 |
|  | AlloSCT | 0.18 | 0.15 - 0.22 | 0.23 | 0.19 - 0.27 |
|  | Relapse | 0.05 | 0.02 - 0.07 | 0.03 | 0.01 - 0.05 |
|  | Relapse (AlloSCT) | 0.02 | 0.01 - 0.04 | 0.02 | 0.00 - 0.03 |
|  | NRM (no CR) | 0.11 | 0.09 - 0.14 | 0.10 | 0.08 - 0.12 |
|  | NRM (CR) | 0.05 | 0.03 - 0.07 | 0.11 | 0.07 - 0.14 |
|  | NRM (AlloSCT) | 0.09 | 0.07 - 0.12 | 0.09 | 0.06 - 0.11 |
|  | RM | 0.16 | 0.13 - 0.20 | 0.14 | 0.10 - 0.17 |
|  | RM (AlloSCT) | 0.16 | 0.13 - 0.20 | 0.11 | 0.08 - 0.14 |

**Table S4**: Transition probabilities from CR within 3 months. Transition probabilities (plus 95% CI) at 24 and 60 months since randomization per treatment arm given that the patient has achieved CR within 3 months since randomization.
AlloSCT: allogeneic stem cell transplantation, CI: confidence interval, CR: complete remission, HR: hazard ratio, NRM: non-relapse mortality, RM: relapse mortality (all mortality taking place after relapse), TP: transition probability.

|  |  | **Standard arm** | | **Clofarabine arm** | |
| --- | --- | --- | --- | --- | --- |
| **Time** | **State** | **TP** | **95% CI** | **TP** | **95% CI** |
| 24 months | CR | 0.25 | 0.20 - 0.30 | 0.27 | 0.22 - 0.32 |
|  | AlloSCT | 0.25 | 0.21 - 0.30 | 0.30 | 0.25 - 0.35 |
|  | Relapse | 0.09 | 0.06 - 0.13 | 0.06 | 0.03 - 0.09 |
|  | Relapse (AlloSCT) | 0.02 | 0.01 - 0.04 | 0.02 | 0.00 - 0.04 |
|  | NRM (CR) | 0.03 | 0.01 - 0.05 | 0.06 | 0.03 - 0.09 |
|  | NRM (AlloSCT) | 0.07 | 0.05 - 0.09 | 0.06 | 0.04 - 0.09 |
|  | RM | 0.14 | 0.10 - 0.18 | 0.12 | 0.09 - 0.16 |
|  | RM (AlloSCT) | 0.14 | 0.10 - 0.17 | 0.10 | 0.07 - 0.12 |
| 60 months | CR | 0.22 | 0.17 - 0.26 | 0.24 | 0.19 - 0.29 |
|  | AlloSCT | 0.20 | 0.16 - 0.24 | 0.25 | 0.20 - 0.30 |
|  | Relapse | 0.06 | 0.03 - 0.09 | 0.04 | 0.01 - 0.06 |
|  | Relapse (AlloSCT) | 0.02 | 0.01 - 0.04 | 0.02 | 0.00 - 0.04 |
|  | NRM (CR) | 0.03 | 0.01 - 0.06 | 0.07 | 0.04 - 0.10 |
|  | NRM (AlloSCT) | 0.10 | 0.07 - 0.13 | 0.09 | 0.06 - 0.12 |
|  | RM | 0.20 | 0.16 - 0.25 | 0.17 | 0.13 - 0.22 |
|  | RM (AlloSCT) | 0.17 | 0.13 - 0.21 | 0.12 | 0.09 - 0.15 |

**Table S5**: Distribution of ELN risk group per treatment arm.

|  | **Standard arm** | | **Clofarabine arm** | |
| --- | --- | --- | --- | --- |
| **ELN risk group** | **N** | **%** | **N** | **%** |
| Favorable | 106 | 26.4 | 85 | 21.6 |
| Intermediate I | 123 | 30.6 | 127 | 32.3 |
| Intermediate II | 83 | 20.6 | 95 | 24.2 |
| Adverse | 90 | 22.4 | 86 | 21.9 |
| Total | 402 | 100 | 393 | 100 |

**Table S6**: Transition probabilities from randomization by ELN 2010 risk group. Transition probabilities (plus 95% CI) at 24 and 48 months since randomization per treatment arm and ELN 2010 risk group.
AlloSCT: allogeneic stem cell transplantation, CI: confidence interval, CR: complete remission, HR: hazard ratio, NRM: non-relapse mortality, RM: relapse mortality (all mortality taking place after relapse), TP: transition probability.

|  |  |  | **Standard arm** | | **Clofarabine arm** | |
| --- | --- | --- | --- | --- | --- | --- |
| **ELN risk** | **Time** | **State** | **TP** | **95% CI** | **TP** | **95% CI** |
| Favorable risk | 24 months | Randomization | 0.00 | 0.00 - 0.01 | 0.01 | 0.00 - 0.02 |
|  |  | CR | 0.50 | 0.41 - 0.59 | 0.58 | 0.48 - 0.68 |
|  |  | AlloSCT | 0.14 | 0.08 - 0.20 | 0.11 | 0.04 - 0.17 |
|  |  | Relapse | 0.17 | 0.10 - 0.24 | 0.06 | 0.01 - 0.12 |
|  |  | Relapse (AlloSCT) | 0.01 | 0.01 - 0.02 | 0.00 | 0.01 - 0.02 |
|  |  | NRM (no CR) | 0.02 | 0.00 - 0.04 | 0.08 | 0.03 - 0.14 |
|  |  | NRM (CR) | 0.03 | 0.00 - 0.06 | 0.10 | 0.04 - 0.16 |
|  |  | NRM (AlloSCT) | 0.02 | 0.00 - 0.04 | 0.01 | 0.01 - 0.03 |
|  |  | RM | 0.09 | 0.04 - 0.14 | 0.03 | 0.00 - 0.06 |
|  |  | RM (AlloSCT) | 0.02 | 0.00 - 0.05 | 0.01 | 0.01 - 0.03 |
|  | 48 months | Randomization | 0.00 | 0.00 - 0.01 | 0.01 | 0.00 - 0.02 |
|  |  | CR | 0.46 | 0.37 - 0.55 | 0.56 | 0.46 - 0.66 |
|  |  | AlloSCT | 0.10 | 0.05 - 0.16 | 0.09 | 0.03 - 0.15 |
|  |  | Relapse | 0.13 | 0.07 - 0.20 | 0.05 | 0.00 - 0.10 |
|  |  | Relapse (AlloSCT) | 0.00 | 0.00 - 0.00 | 0.00 | 0.00 - 0.00 |
|  |  | NRM (no CR) | 0.02 | 0.00 - 0.04 | 0.08 | 0.03 - 0.14 |
|  |  | NRM (CR) | 0.03 | 0.00 - 0.06 | 0.11 | 0.05 - 0.18 |
|  |  | NRM (AlloSCT) | 0.06 | 0.02 - 0.10 | 0.03 | 0.00 - 0.07 |
|  |  | RM | 0.16 | 0.09 - 0.23 | 0.06 | 0.01 - 0.11 |
|  |  | RM (AlloSCT) | 0.03 | 0.00 - 0.06 | 0.01 | 0.01 - 0.04 |
| Intermediate I risk | 24 months | Randomization | 0.00 | 0.00 - 0.00 | 0.00 | 0.00 - 0.00 |
|  |  | CR | 0.07 | 0.03 - 0.10 | 0.20 | 0.13 - 0.26 |
|  |  | AlloSCT | 0.27 | 0.19 - 0.35 | 0.35 | 0.27 - 0.43 |
|  |  | Relapse | 0.04 | 0.01 - 0.08 | 0.05 | 0.02 - 0.09 |
|  |  | Relapse (AlloSCT) | 0.04 | 0.00 - 0.07 | 0.02 | 0.01 - 0.06 |
|  |  | NRM (no CR) | 0.16 | 0.10 - 0.22 | 0.07 | 0.03 - 0.10 |
|  |  | NRM (CR) | 0.07 | 0.01 - 0.12 | 0.07 | 0.01 - 0.12 |
|  |  | NRM (AlloSCT) | 0.06 | 0.02 - 0.10 | 0.06 | 0.02 - 0.10 |
|  |  | RM | 0.15 | 0.09 - 0.20 | 0.12 | 0.07 - 0.17 |
|  |  | RM (AlloSCT) | 0.15 | 0.09 - 0.21 | 0.06 | 0.01 - 0.10 |
|  | 48 months | Randomization | 0.00 | 0.00 - 0.00 | 0.00 | 0.00 - 0.00 |
|  |  | CR | 0.04 | 0.01 - 0.07 | 0.15 | 0.09 - 0.21 |
|  |  | AlloSCT | 0.23 | 0.15 - 0.30 | 0.32 | 0.24 - 0.40 |
|  |  | Relapse | 0.03 | 0.00 - 0.05 | 0.04 | 0.00 - 0.08 |
|  |  | Relapse (AlloSCT) | 0.03 | 0.00 - 0.07 | 0.02 | 0.01 - 0.05 |
|  |  | NRM (no CR) | 0.16 | 0.10 - 0.22 | 0.07 | 0.03 - 0.10 |
|  |  | NRM (CR) | 0.07 | 0.02 - 0.12 | 0.08 | 0.02 - 0.13 |
|  |  | NRM (AlloSCT) | 0.07 | 0.03 - 0.12 | 0.08 | 0.03 - 0.12 |
|  |  | RM | 0.19 | 0.13 - 0.25 | 0.17 | 0.11 - 0.24 |
|  |  | RM (AlloSCT) | 0.18 | 0.11 - 0.24 | 0.07 | 0.03 - 0.12 |
| Intermediate II risk | 24 months | Randomization | 0.00 | 0.00 - 0.01 | 0.01 | 0.01 - 0.03 |
|  |  | CR | 0.17 | 0.09 - 0.24 | 0.06 | 0.02 - 0.10 |
|  |  | AlloSCT | 0.33 | 0.23 - 0.42 | 0.39 | 0.29 - 0.48 |
|  |  | Relapse | 0.06 | 0.00 - 0.13 | 0.03 | 0.00 - 0.06 |
|  |  | Relapse (AlloSCT) | 0.01 | 0.01 - 0.03 | 0.01 | 0.01 - 0.03 |
|  |  | NRM (no CR) | 0.08 | 0.03 - 0.13 | 0.08 | 0.03 - 0.12 |
|  |  | NRM (CR) | 0.07 | 0.02 - 0.12 | 0.13 | 0.06 - 0.19 |
|  |  | NRM (AlloSCT) | 0.07 | 0.02 - 0.13 | 0.07 | 0.02 - 0.12 |
|  |  | RM | 0.11 | 0.04 - 0.18 | 0.13 | 0.07 - 0.19 |
|  |  | RM (AlloSCT) | 0.10 | 0.04 - 0.16 | 0.09 | 0.04 - 0.15 |
|  | 48 months | Randomization | 0.00 | 0.00 - 0.01 | 0.01 | 0.01 - 0.03 |
|  |  | CR | 0.15 | 0.08 - 0.23 | 0.05 | 0.01 - 0.09 |
|  |  | AlloSCT | 0.29 | 0.20 - 0.39 | 0.35 | 0.26 - 0.44 |
|  |  | Relapse | 0.04 | 0.01 - 0.09 | 0.01 | 0.01 - 0.03 |
|  |  | Relapse (AlloSCT) | 0.01 | 0.01 - 0.03 | 0.01 | 0.01 - 0.03 |
|  |  | NRM (no CR) | 0.08 | 0.03 - 0.13 | 0.08 | 0.03 - 0.12 |
|  |  | NRM (CR) | 0.08 | 0.03 - 0.13 | 0.13 | 0.07 - 0.20 |
|  |  | NRM (AlloSCT) | 0.09 | 0.03 - 0.14 | 0.08 | 0.03 - 0.14 |
|  |  | RM | 0.14 | 0.06 - 0.21 | 0.15 | 0.09 - 0.22 |
|  |  | RM (AlloSCT) | 0.12 | 0.05 - 0.19 | 0.12 | 0.06 - 0.18 |
| Adverse risk | 24 months | Randomization | 0.00 | 0.00 - 0.00 | 0.00 | 0.00 - 0.00 |
|  |  | CR | 0.02 | 0.00 - 0.03 | 0.02 | 0.00 - 0.04 |
|  |  | AlloSCT | 0.22 | 0.14 - 0.30 | 0.19 | 0.11 - 0.27 |
|  |  | Relapse | 0.02 | 0.01 - 0.04 | 0.02 | 0.02 - 0.06 |
|  |  | Relapse (AlloSCT) | 0.05 | 0.00 - 0.09 | 0.03 | 0.01 - 0.07 |
|  |  | NRM (no CR) | 0.20 | 0.13 - 0.27 | 0.19 | 0.12 - 0.25 |
|  |  | NRM (CR) | 0.02 | 0.01 - 0.05 | 0.11 | 0.04 - 0.17 |
|  |  | NRM (AlloSCT) | 0.12 | 0.06 - 0.18 | 0.11 | 0.05 - 0.17 |
|  |  | RM | 0.09 | 0.04 - 0.14 | 0.11 | 0.05 - 0.18 |
|  |  | RM (AlloSCT) | 0.27 | 0.18 - 0.35 | 0.23 | 0.15 - 0.31 |
|  | 48 months | Randomization | 0.00 | 0.00 - 0.00 | 0.00 | 0.00 - 0.00 |
|  |  | CR | 0.02 | 0.00 - 0.03 | 0.02 | 0.00 - 0.04 |
|  |  | AlloSCT | 0.16 | 0.09 - 0.23 | 0.14 | 0.07 - 0.21 |
|  |  | Relapse | 0.01 | 0.01 - 0.03 | 0.01 | 0.01 - 0.04 |
|  |  | Relapse (AlloSCT) | 0.02 | 0.01 - 0.05 | 0.01 | 0.01 - 0.04 |
|  |  | NRM (no CR) | 0.20 | 0.13 - 0.27 | 0.19 | 0.12 - 0.25 |
|  |  | NRM (CR) | 0.02 | 0.01 - 0.05 | 0.11 | 0.04 - 0.17 |
|  |  | NRM (AlloSCT) | 0.15 | 0.08 - 0.22 | 0.14 | 0.07 - 0.20 |
|  |  | RM | 0.09 | 0.04 - 0.15 | 0.12 | 0.06 - 0.18 |
|  |  | RM (AlloSCT) | 0.32 | 0.23 - 0.41 | 0.27 | 0.18 - 0.35 |

**Figure S1**: *Overall survival since randomization. Updated result from Löwenberg et al (Blood 2017) with median follow-up of patients still alive of 72 months (range 10 - 108 months).*

*
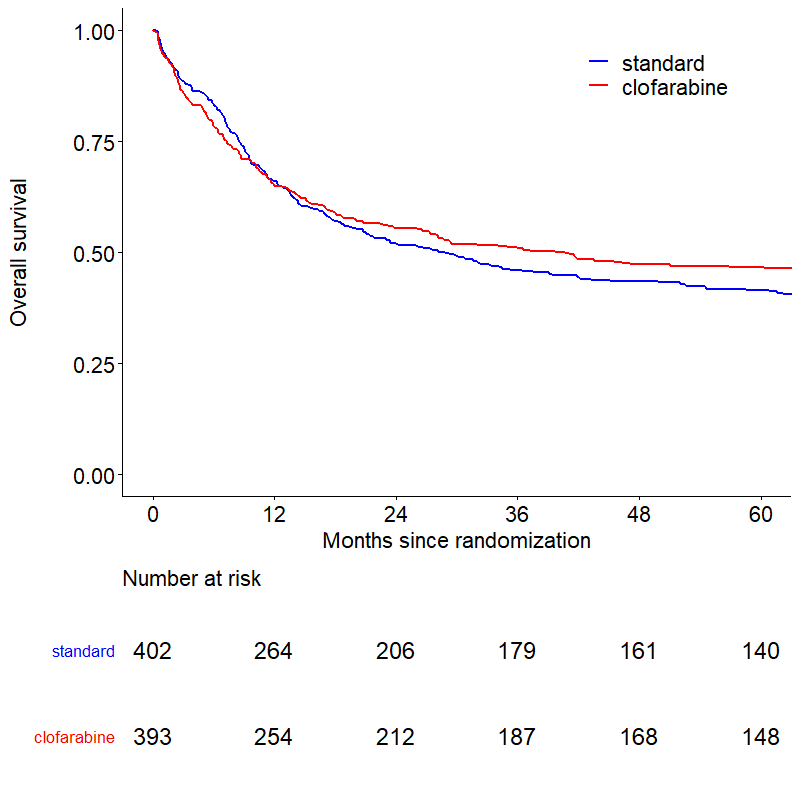
*

**Figure S2**: Cumulative incidence curves of relapse and non-relapse mortality of complete responders: cumulative incidence curves of relapse (top) and non-relapse mortality (bottom) of patients achieving complete remission per treatment arm.
CR: complete remission, NRM: non-relapse mortality.


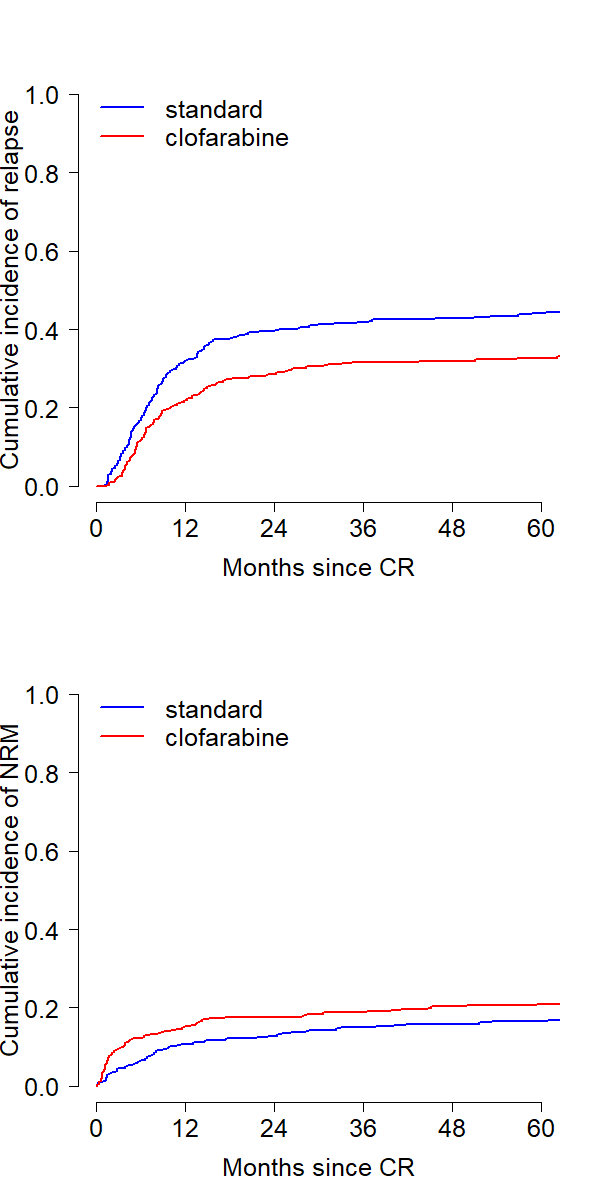


**Figure S3**: Cumulative incidence curve of MRD- per treatment arm, where progression and death were considered as competing risks.
MRD-: measurable residual disease negativity.


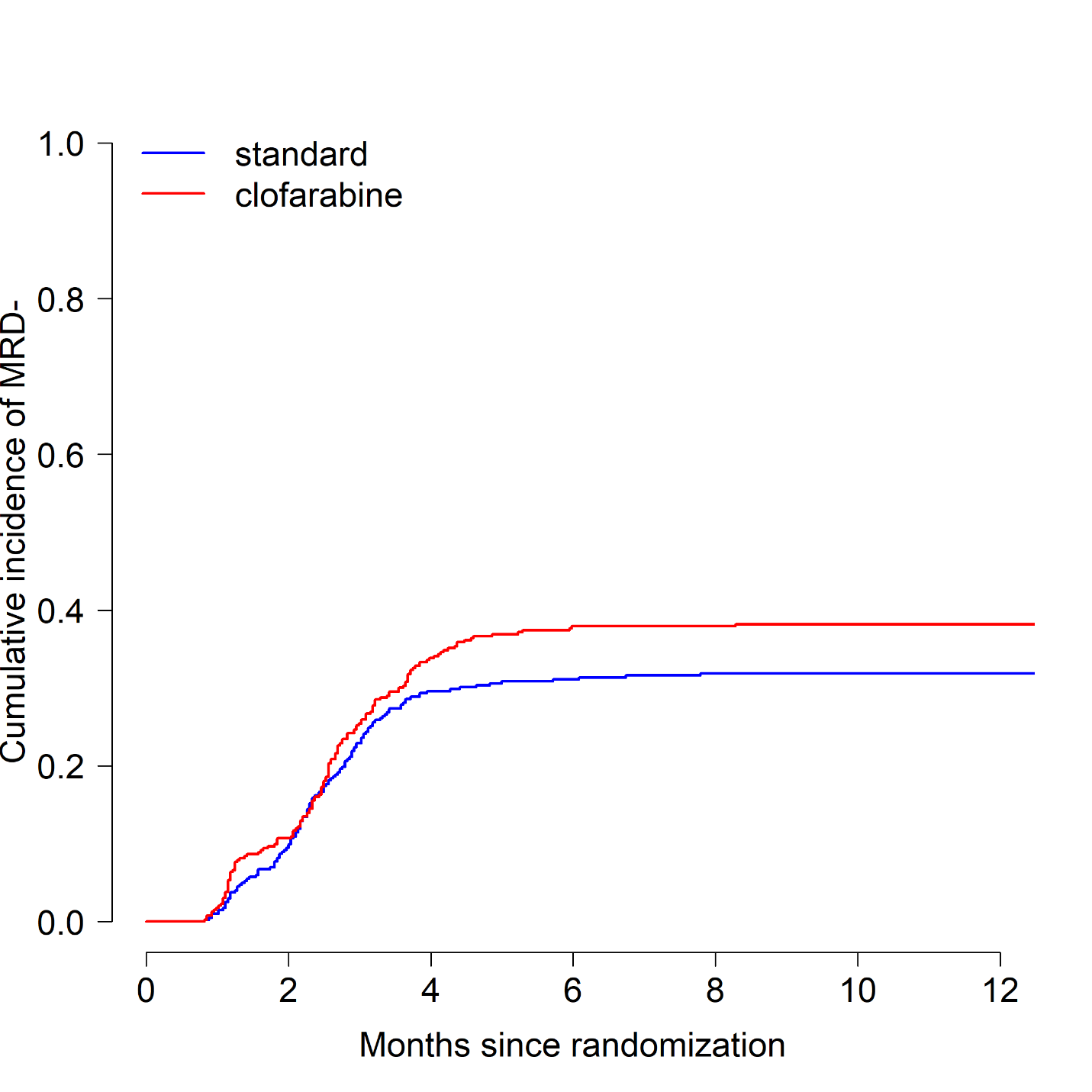


**Figure S4**: Transition probabilities to all states from randomization for three ELN risk groups per treatment arm: Semi-parametric estimates of the transition probabilities to all states from randomization for the Favorable risk group (row 1), Intermediate II risk group (row 2), and Adverse risk group (row 3). The transition probabilities for the Intermediate-I risk group are presented in Figure 6.
AlloSCT: allogeneic stem cell transplantation, CR: complete remission, NRM: non-relapse mortality, RM: relapse mortality (all mortality taking place after relapse).


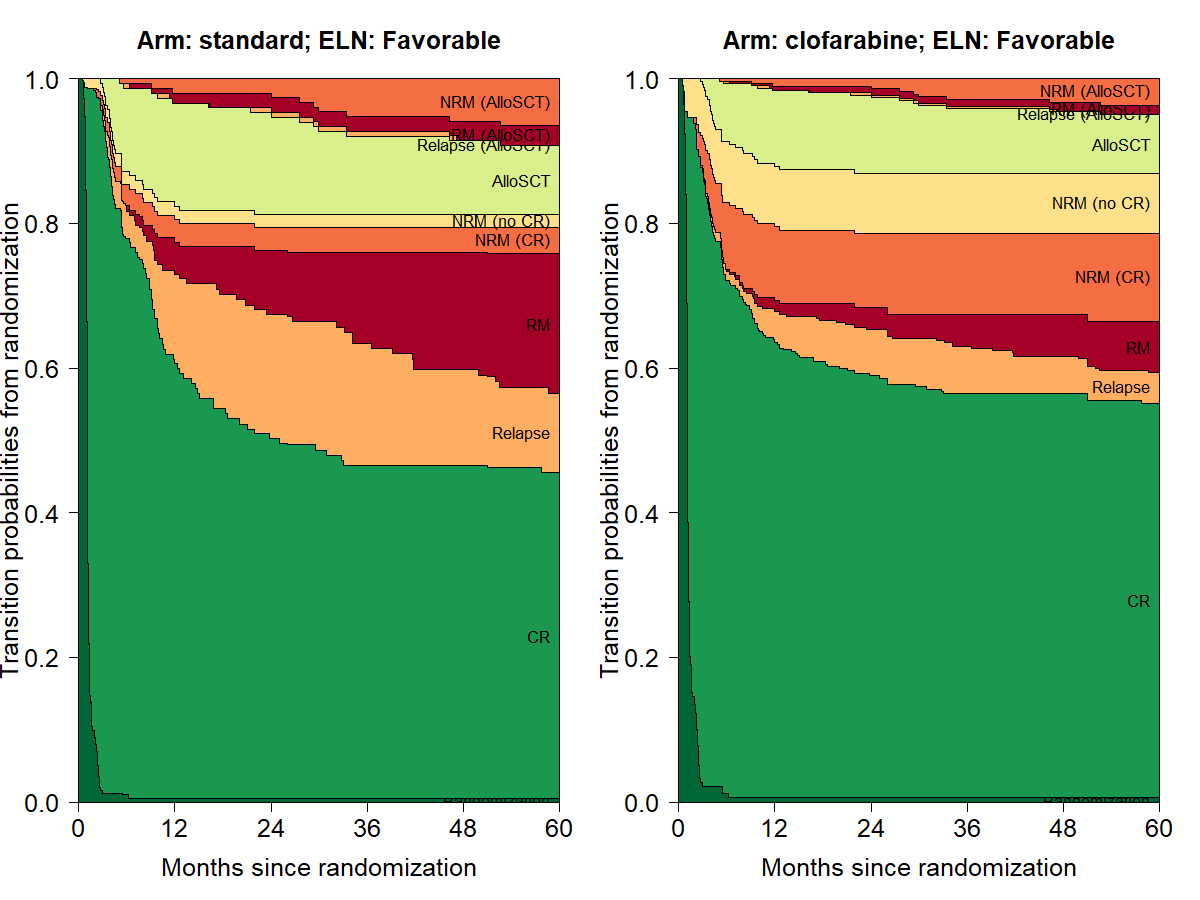


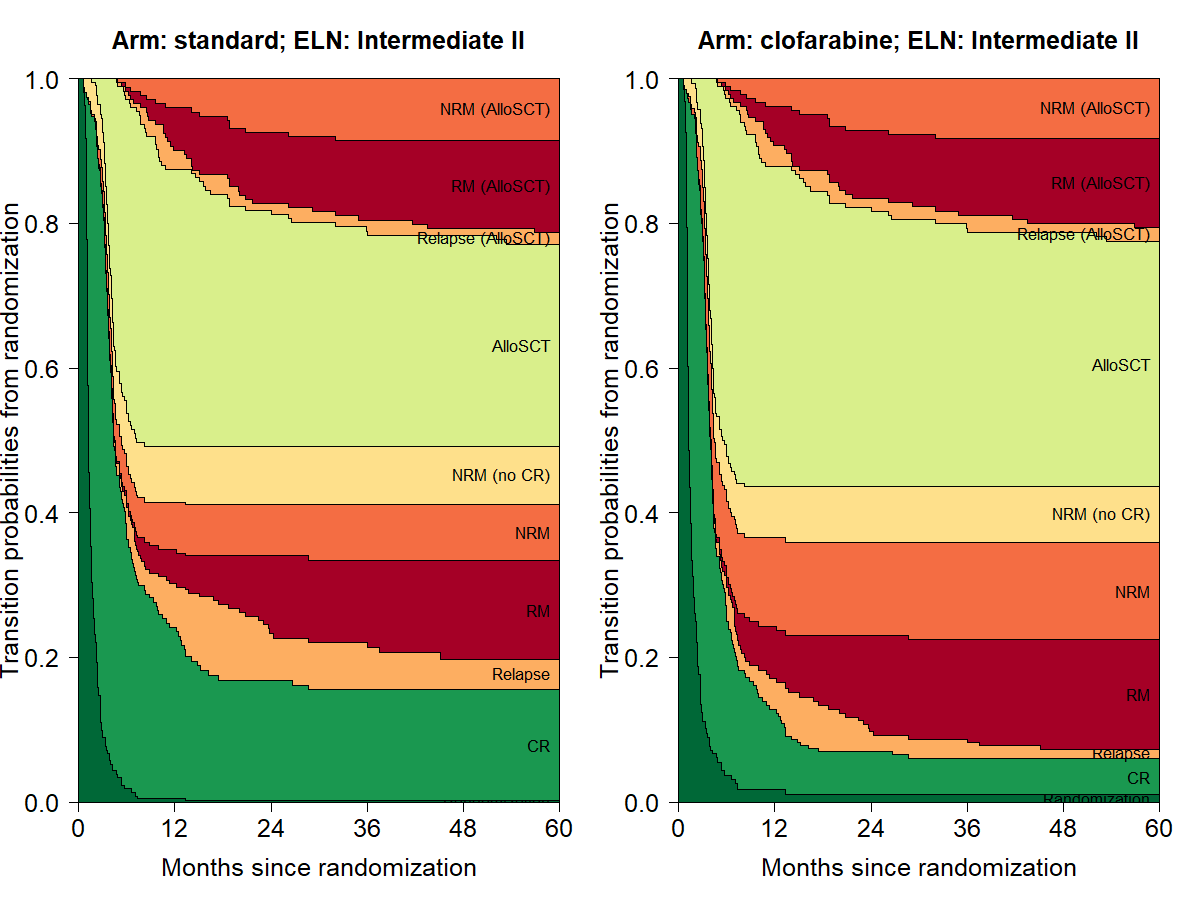


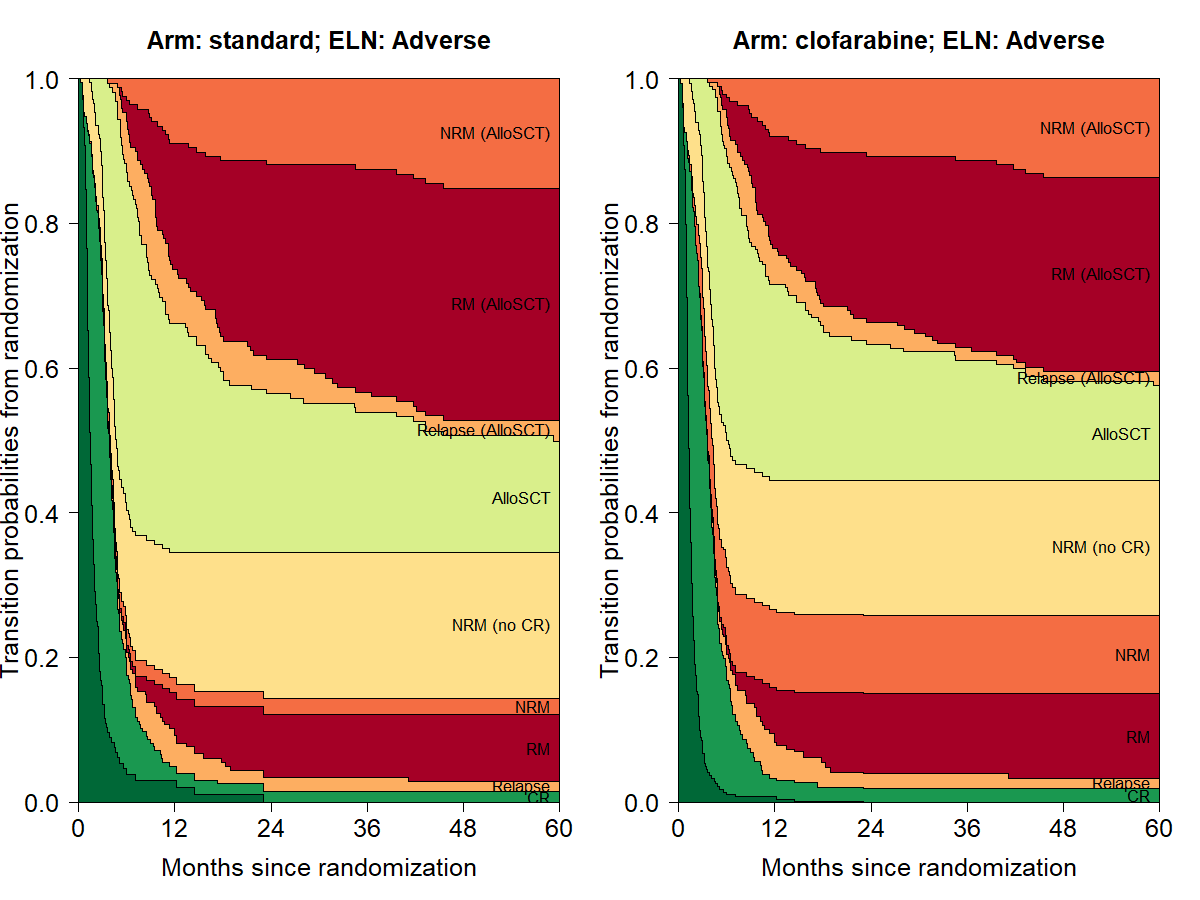

Supplement: Supplementary file 1 — Supplementary Material [file CAM4-11-630-s001.docx]
